# Demystification of animal symmetry: symmetry is a response to mechanical forces

**DOI:** 10.1186/s13062-017-0182-5

**Published:** 2017-05-17

**Authors:** Gábor Holló

**Affiliations:** Institute of Psychology, University of Debrecen, H-4002 Debrecen, P.O. Box 400, Hungary

**Keywords:** Animal symmetry, Body plan, Radial symmetry, Bilateral symmetry, Mechanical forces, Morphogenesis, Evolutionary constraints

## Abstract

**ᅟ:**

Symmetry is an eye-catching feature of animal body plans, yet its causes are not well enough understood. The evolution of animal form is mainly due to changes in gene regulatory networks (GRNs). Based on theoretical considerations regarding fundamental GRN properties, it has recently been proposed that the animal genome, on large time scales, should be regarded as a system which can construct both the main symmetries – radial and bilateral – simultaneously; and that the expression of any of these depends on functional constraints. Current theories explain biological symmetry as a pattern mostly determined by phylogenetic constraints, and more by chance than by necessity. In contrast to this conception, I suggest that physical effects, which in many cases act as proximate, direct, tissue-shaping factors during ontogenesis, are also the ultimate causes – i.e. the indirect factors which provide a selective advantage – of animal symmetry, from organs to body plan level patterns. In this respect, animal symmetry is a necessary product of evolution. This proposition offers a parsimonious view of symmetry as a basic feature of the animal body plan, suggesting that molecules and physical forces act in a beautiful harmony to create symmetrical structures, but that the concert itself is directed by the latter.

**Reviewers:**

This article was reviewed by Eugene Koonin, Zoltán Varga and Michaël Manuel.

## Introduction

Symmetry is a frequent pattern in nature, often perceived as a source of beauty, and is also a salient property of animal body plans. The concept of the body plan can be defined as an ontogenetic pattern-organising algorithm, thanks to which the body develops in a specific order. The two main symmetries that can be observed in the animal body plan are radial and bilateral (for a description of the diverse basic animal symmetries see [1, 2]). Symmetrical biological patterns enchant the human mind, yet a comprehensive explanation for symmetry in biology is lacking. It is thought that the symmetry which appears at high organisational levels, such as in large organisms like animals, is a major consequence of historical (phylogenetic) contingency [3], and is due more to chance than to necessity [4]. I challenge these views proposing that animal symmetry is mostly shaped by mechanical forces, and as such, it is a necessary pattern in animal evolution. In this paper, the factors that directly shape biological patterns will be referred to as direct or proximate causes, while the factors which give a selective advantage to the given form – i.e. they explain what that form is good for – will be termed as indirect or ultimate causes.

It is now widely recognised that the evolution of animal form is mainly caused by the changes in the regulatory genes of the genome [3, 5–12]. These act in a coordinated fashion, in hierarchically organised networks called gene regulatory networks (GRNs) [6–9]. The GRNs determine which protein-coding genes will be transcribed, when and where in the body this transcription will occur, and what quantity of gene-product will be generated. The GRNs are modular [6–9], and they consist of subsystems which are mosaic in terms of evolutionary age and phylogenetic relationships [2, 8]; consequently, GRNs are regarded as historically, structurally and functionally mosaic systems [8]. In this view, in terms of genetic programs, the difference between the establishment of the basic geometrical features of the body plan, the specification of progenitor fields for developing organs, and the formation of tissue-level details, is only a difference in the timing of subsequently activated GRN modules. In accordance with these general and basic properties of GRNs, it has recently been proposed that the determination of the symmetries in diverse levels of the body plan should also be regarded as a question of a different timing, not as the manifestation of a real hierarchical relationship [2, 13] (hierarchy is defined here as the capability of a sub-program to directly control or overwrite another sub-program). In this view, it can be said that the overall symmetry of the body plan is not the only symmetry of the animal, since the symmetries of minor body parts also have to be taken into account when speaking about body plan symmetry.

Regarding the symmetry properties of the animal body, it can be seen that the overall radial symmetry of cnidarians is combined with regional level bilateral symmetry (such as that of the rhopalia, the manubrian arms, the gastric pouches, and the pharynx in the anthozoan polyps); and, similarly, the overall bilateral body symmetry of bilaterians is combined with regional radial symmetry (such as that of the eye balls, and the biological tubes of the circulatory, respiratory, urogenital and glandular conducting systems). Thus, based on theoretical considerations regarding the functioning of the GRNs described above, it has been suggested that the animal body can be regarded as a flexible system in terms of symmetry, capable of constructing either bilateral or radial symmetry [2, 13], be they manifested either in the general body plan or in infraindividual structures. It also has been proposed that the major causes behind the existence of symmetrical structures are functional constraints, given the fact that the symmetry of anatomical structures is associated with strong functionality [2].

GRNs function embedded in a system involving the dynamic exchange of molecular information actuated through morphogen gradient formation and cell–surface contacts. Morphogens are diffusible molecules which govern the pattern formation of tissues during morphogenesis. Several morphogens which are responsible for the formation of the symmetrical body – such as Wnt and bone morphogenetic protein (BMP) –, have been characterised (for an overview on morphogens see [2] and the references therein). Remarkably, mathematical modelling has suggested that merely by coupling two signalling pathways acting in epithelial morphogenesis, under certain parameters the process “automatically” leads to the formation of very basic body plans with either radial or bilateral symmetry [14] (see also [1]). This indicates that the basic molecular organisation required for building any of the two symmetries is relatively simple.

However, growth is a mechanical process, and whereas the role of morphogens is indisputable, they cannot be expected to act alone [15–18]. Simply put, genes and GRNs are not everything. Such a reductionist view neglects the important fact that living organisms, too, function in an environment where the laws of physics are as valid as in the non-living world, so they are under the influence of the same basic architectural principles (described by the fundamental laws of physics) that shape the non-living natural world [19]. Thus, tracing everything back to molecules while searching for the ultimate causes of biological processes can be misleading because this kind of approach omits other factors without which the molecular systems could not work properly. Genes constitute the plan for building the body, but molecules can only act in an appropriate set of physical circumstances. Since morphogens act in a physical entity – the developing tissue –, tissue morphogenesis should be regarded as a process which is under genetic control but which also occurs by the action of mechanical forces [15–26]. Mechanical forces, in contrast to local effects, may also act globally, which can be important while organs develop to achieve their correct sizes and shapes [16]. Since cells are interconnected, cell proliferation and shape changes potentially affect the whole tissue or organ, inducing mechanical stress, even when they are local phenomena [16]. Moreover, the physical environment may not only function as the matrix in which the biological processes occur, but can also be the guiding factor which drives the molecules and cells to act both during the formation of a given tissue and during the functioning of the anatomical structure (see also [15, 20, 23, 27–29]). I suggest that in the case of most symmetrical biological structures this is exactly what happens. Symmetry is a response in the geometry of the “living matter” to physical forces.

## Mechanical forces and morphogenesis

### Influence of mechanical forces on morphogenetic processes

That the structure and form of the animal body and body parts is often shaped by mechanical forces is not a new observation in developmental biology, and it has ever growing theoretical and experimental support. More than half a century ago, Coulombre reported that the development of the correct eye size in chickens was influenced by tensile forces on the embryonic eye wall [30]. Similarly, Coulombre and co-authors suggested that the pigmented epithelium of chicken embryonic eyes increased in area in response to tensile forces acting in its plane [31]. Later on, Desmond and Jacobson pointed out that the correct enlargement and shaping of the chick embryonic brain was dependent on the mechanical force produced by cerebrospinal fluid pressure [32]. In the twenty-first century, several similar cases have been described. The role of mechanical forces has been reported in shaping skeletal structures such as the sophisticated skeleton of the hexactinellid sponge *Euplectella* [33] and the interesting, square-shaped tail of the seahorse [34]. Mechanical forces have been implicated in the correct morphogenesis of zebrafish glomeruli [35], heart [36], gut [37], nephron [38], intersegmental vessels [39] and brain ventricles [40], as well as in the process of normal haematopoiesis [41]; in the morphogenesis of the *Caenorhabditis elegans* vulva [42] and excretory canal [43]; in the *Drosophila* wing imaginal disc [16]; in the development of the rat lung [44, 45] and bone [46]; and in the development of the chick heart [47], and of the neurons of the locust [48]. Similarly, mechanical forces have been described as important regulatory factors in the correct development of the mouse lung [45, 49, 50], mammary gland [51], lymphatic vasculature [52], and neurons [53], in the remodelling of yolk sac vessels [54], in normal angiogenesis [55], joint formation [56] and haematopoiesis [41, 57], as well as in human angiogenesis [55]. Mechanical stress produced only by tissue form has been shown to induce spatial patterning of cell proliferation during tissue morphogenesis [20]. Theoretical modelling, too, has supported the idea that epithelial morphogenesis is organised by a complex interplay between mechanical forces and signalling pathways [58]. Similarly, a thorough study highlighted the importance of mechanical forces in epithelial tubulogenesis, showing that morphogenesis of tubes was initiated and maintained by a mechanical interaction between the cells and collagen fibres of the extracellular matrix [27], thus underscoring the significance of mechanical forces enriching the conventional concept which considers mainly – or only – the action of genes and morphogens [17, 19, 21, 27, 29]. Remarkably, a recent study has revealed the role of whole-embryo-scale mechanical forces during the gastrulation process in *Drosophila* [59]. It also has been reported that the morphogenesis of the looped vertebrate gut is explained by simple mechanical forces caused by the differential growth of the gut tube and the anchoring mesenteric sheet, and by the elastic and geometric properties of their tissues [60]. Likewise, it has been shown that mechanical forces acting between the different tissue layers of the developing gut account for the process in which the intestinal villi are generated [61]. Although it was the chick villification that was described, the theoretical considerations also seem to be applicable to a variety of other animals [61]. Tallinen and co-authors have shown that similar mechanical forces underlie the process of gyrification in the mammalian brain, including the human fetal brain [62, 63]. (Interestingly, a theoretical mechanical model of the convolutional development of the brain has existed for more than 40 years [64]). Based on results of in vitro stem cell research, relatively simple local mechanical rules have been proposed as drivers of the complex phenomenon of optic cup self-organisation [28]. In a wide-ranging article, Banavar et al. have recently shown that despite the enormous differences in the shape of vascular plants and bilaterian animals, the processes of transformation, transport, and exchange of matter and energy impose fundamental physical constraints on their body design [65].

Extensive work has been carried out on the interplay between mechanical forces and cellular–subcellular processes during tissue morphogenesis (e.g. [20–22, 24–26, 66–69]), but it cannot necessarily be expected that the shape and symmetry of larger anatomical structures – being at a higher level of biological organisation – can be deduced simply from these kinds of effects. So, although supracellular-level growth processes are clearly influenced by cellular-level mechanical effects (and vice versa), this topic will not be developed further here.

The above-cited examples are far from exhaustive, yet they indicate that the physical constraints on the development of a variety of anatomical patterns may act much more pervasively than generally recognised. These examples – several of which describe symmetrical structures – have thus highlighted that – speaking generally about morphogenesis – the conceptions that view morphogenetic phenomena as processes directed strictly by genes and morphogenes alone must be abandoned, and substituted by a view which also includes the role of mechanical forces.

### Mechanical forces and the formation of symmetrical internal anatomical structures

Radial symmetry is a pervasive pattern in internal anatomical structures, since the innumerable biological tubes which constitute transport systems in the animal body, are characterized by this symmetry [2]. Biological tubes are generally small when they are generated, and later grow by one or two orders of magnitude to attain definitive sizes [70]. This growth is accompanied by the rearrangement of cells which can also proliferate, e.g. [70, 71] (on the molecular background of tubulogenesis see for example [2] and the references therein). On the one hand, radial signal gradients can be expected to account for the radial growth of symmetrical structures. For instance, it has been proposed that the radial construction of the pulmonary artery wall in mice is orchestrated by an ensemble of radially diffusing factors [72]. On the other hand, mechanical effects are also expected to regulate the shape of tubular organs during growth. Indeed, as the number of examples grows, it seems even clearer that a crucial mechanism for the maintenance of the radial symmetry of biological tubes is that of mechanical forces acting from the inside of the lumen: tension caused by liquid secretion into the lumen has already been implicated during tube expansion (reviewed in [70, 71]). For instance, luminal hydrostatic pressure has been shown to be responsible for the lumen extension of the *C. elegans* excretory tube [43], and the maintenance of the newly formed lumen has also been demonstrated to be dependent on hydrostatic pressure produced by blood flow in zebrafish embryonic intersegmental vessels [39]. Similarly, intraluminal chitin matrix has also been described as mechanically driving luminal expansion in *Drosophila* trachea [71]. Remarkably, this type of mechanical shaping of tubes by luminal extracellular matrices may also function in other developing epithelial organs of *Drosophila*, and also of other organisms such as chicken and *C. elegans* (reviewed in [71]). According to Laplace’s law, in a cylinder with internal pressure, the circumferential surface tension is always greater than the axial surface tension (as in the example of the over-boiled hot dog sausage; [71]), so it is very probable that this force largely contributes to the enlargement of the tube. Nevertheless, the problem of whether this is a general mechanism for tube growth remains unclear (for details on molecular mechanisms see [71]). Thus, based on the above-mentioned reasons, it can be supposed that the maintenance of radial symmetry in growing organs is largely determined by mechanical forces which thus serve as an immediate, direct means of the building of radial symmetry.

The exact mechanisms by which internal bilateral symmetry builds have been in part elucidated, although several aspects remain unclear. For example, it has been reported that the placement of the node and the notochord along the plane of bilateral symmetry in mice requires the proper interaction of the extracellular matrix protein fibronectin and the cellular receptor integrin α5β1, probably necessary for generating and/or maintaining mechanical forces between cells [73]; however, the whole process of the formation of the symmetry plane, probably also including the factors which direct the interaction of the molecules mentioned, remains elusive. In another example regarding the formation of internal bilateral symmetry – more specifically, the establishment of the symmetry plane of the neural rod in Zebrafish embryos, a key element in the formation of bilateral symmetry –, many details have been explored, including the role of the polarity protein Pard3 in midline-crossing cell divisions [74], that of the orientation of these stereotypical divisions [75] controlled by Scribble [76], and the complex cellular rearrangement by which cells from the two sides stop at the precise geometrical midline [77]. How the cells exactly sense the midline and how they stop there, however, remains a mystery [77]. With the elucidation of the mechanism of this process, the key to the maintenance of bilateral symmetry by morphogens or other mechanisms during growth could also be discovered. Remarkably, Žigman and co-authors [76] showed that the molecular control on the mitotic spindle orientation during the midline-crossing cell divisions that give rise to the bilaterally arranged neural tube tissue of zebrafish is not exclusive, and they proposed that a cellular community effect stemming from external physical forces may also play an important role in the process.

All the examples mentioned above only describe the direct causes that shape symmetrical structures, that is to say, how physical forces help them form. However, the answer to the bigger question of what the indirect causes of the two main symmetries are, is still missing. On the level of internal anatomical structures, the radial symmetry of the many types of biological tubes is explained by the balanced distribution of transported material [2], but internal bilateral symmetry apparently has no such obvious direct benefits; it rather seems to be the necessary internal concomitant of an overall bilateral body symmetry (on the presumptive evolutionary advantage of the internal, bilaterally symmetrical structures of cnidarians, see ref. [78]). Turning our attention towards the whole body and asking about the indirect, ultimate causes of symmetry, the answers invoke mechanical forces again.

### Mechanical forces and the overall body symmetry: the establishment of symmetry in the animal body and the indirect causes of body plan symmetry

**Fig. 1:**
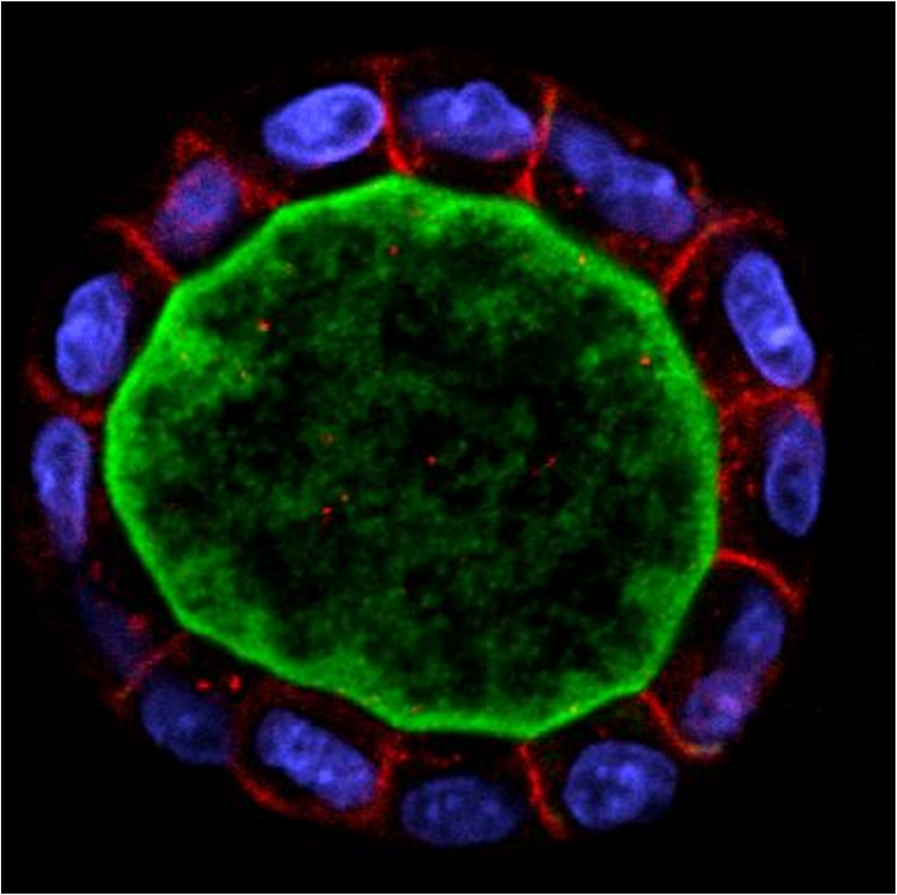
Confocal section of a Madin-Darby canine kidney (MDCK) cyst grown in Matrigel. Cells form a spherical cyst in the first step of renal tubulogenesis (apical membrane and lumen: *green*; nucleus: *blue*; basolateral membrane: *red*; staining and related information on cyst and markers used can be obtained from [97]). Photo courtesy of Sang-Ho Kwon and Keith Mostov

To further explore the deep connection between mechanical forces and symmetry, it seems to be useful to observe how symmetry is established in the first place. Overall body symmetry arises at the beginning of development, from the original spherical symmetry which forms by the physical effects of the microscopic world. In this realm, before tissue stabilisation, aggregates of motile and mutually adhesive cells essentially behave as liquids, and their shape changes are governed by surface tension via the diminution in the adhesive-free energy of the cell population (that is, the maximisation of adhesive bonding) [21, 79] and the actomyosin-dependent cell-cortex tensions [21, 80]. With the formation of the blastula, the spherical symmetry that is established is a simple reaction to the physical environment: cells spontaneously take a spherical form, minimising their total surface area, and this shape is also the simplest geometrical arrangement which responds to equally distributed forces (given for example by the fluid pressure from the inside of the blastula). Importantly, this also seems to happen when the primordia of radially symmetrical internal structures are generated, such as in the case of the cyst formation which precedes renal tubulogenesis (Fig. 1). Later on, in the developing embryo, the overall symmetry is determined by the establishment of polarity axes in the globally spherical set of cells that precedes the embryo, thus causing the breaking of a more perfect symmetry. Creating one polarity axis in a spherical structure, leads to radial symmetry; with the creation of a second axis, bilateral symmetry is determined. The establishment of polarity axes primarily occurs through the action of diffusible morphogen molecules. This process is accompanied, and also effectuated, by morphogenetic events such as the formation of germ layers: in radially symmetrical taxa, the ectoderm and endoderm are generated, to which the mesoderm and the coelom are added in bilateral animals. Thus, nature adopts an elegant way to establish radial or bilateral body symmetry: in the first step, the most perfect – spherical – symmetry is generated, and then it is “flawed” to create radial or bilateral symmetry. Interestingly, mechanical forces have recently been described as also guiding the first breaking of the spherical symmetry of cysts, a process which occurs in order to generate tubes, as observable during the development of biliary ducts in the liver [81, 82].

What are these body symmetries good for? I propose that mechanical forces, besides being able to work as proximate, tissue-shaping factors, also account for the indirect purposes of radial and bilateral body plan symmetry. To try to understand these purposes, I think it is worth approaching them through the role of locomotion, beginning with an examination of bilaterality.

**Fig. 2:**
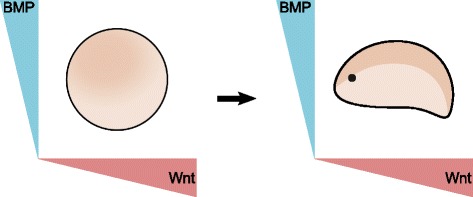
The bilaterally symmetrical body plan of most animals is generated by two, perpendicularly acting diffusible morphogen gradients: Wnt and BMP. The figure has been inspired by Fig. five of [5]. Note that the BMP gradient is oriented in the opposite direction in chordates

**Fig. 3:**
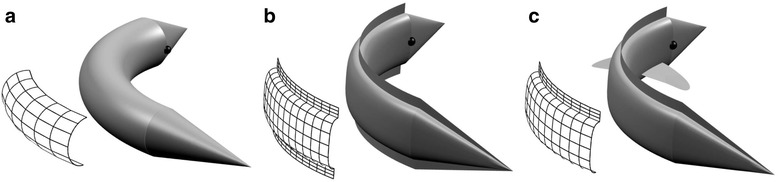
Radially (**a**), biradially (**b**) and bilaterally (**c**) symmetrical bodies with the projection of pushing surfaces created in a watery environment. Grids indicate the approximate magnitude of resistance necessary to produce turning forces

Bilateral symmetry is a major enigma in biology. This symmetry is generated by setting up an anteroposterior (AP) and a dorsoventral (DV) polarity axis during gastrulation. The general mechanism behind the determination of these axes in most animals is the action of two perpendicularly diffusing morphogen gradients, Wnt and BMP (Fig. 2) [5, 83, 84]. The mirror symmetrical pattern of the body plan of Bilateria has attracted much attention in biological textbooks, but a comprehensive theory that could fully and precisely explain the evolutionary significance of bilaterality is still missing. Bilateral symmetry had long been associated with directed locomotion (e.g. [85–87]), although how precisely an efficient directed locomotion could account for bilateral symmetry, has long remained unclear. To date, the most comprehensive idea which explains how directed locomotion is favoured by bilateral symmetry comes from a theoretical paper [88], which argued that bilateral symmetry is favourable for manoeuvrable locomotion in the macroscopic world (in which inertial forces dominate over viscous forces, i.e., in the high Reynolds numbers’ realm (e.g. [89]), because bilateral is the only type of symmetry which is streamlined in only one direction while being non-streamlined in others. Thanks to this, the bilateral body can move forward very efficiently, and it can also produce a greater pushing force in sideways directions compared to other streamlined symmetry types, thus ensuring the maximisation of turning forces [88] (Fig. 3). This is also helped by the bilaterally positioned appendages with which the bilateral body can further augment its sideways resistance without losing too much on skin friction, hence effectuating a kind of trade-off between the slowing effect due to the increased surface and the gained pushing force stemming from resistance (picture the body of a fish, for example). This clearly cannot be optimised to such an extent in a radially symmetrical body in which the theoretical, radially arranged appendages, besides offering the possibility to turn in many directions without twisting the body, would augment the surface and so skin friction superfluously, because the appendages which did not actually work in the given body movement would represent an unnecessary burden (or would have to be instantaneously retracted and stuck out, continuously). The process is best carried out with the use of bilaterally ordered appendages combined with body twists and turns.

To complete the picture, it is important to mention the role of gravity in the determination of dorsoventral polarity [1, 90]. To produce sideways turning forces it is enough to have a laterally flattened body (Fig. 3B), i.e. biradial symmetry. However, in dimensions characterised by even greater Reynolds numbers, the viscosity of the fluid will be not enough to hold the body, and hydrostatic pressure will not be able to fully counteract gravity. In this realm, the dorsoventral polarisation, which produces a different profiling of the dorsal and ventral sides of the body, and, most importantly, of the appendages, will help to produce a lifting force. This dorsoventral polarisation leads to the advent of the second polarity axis, thus reducing the number of the two symmetry planes of a biradial body to one, generating a bilaterally symmetrical body. Later on in evolution, bilaterally symmetrical locomotor apparati proved to be useful both on land, where locomotion essentially occurs in a 2D environment, requiring the body to go directly and to turn left or right, and in the air, where the 3D locomotion is similar to that found in water, and to overcome gravity, large surface wings counterbalance the lack of hydrostatic pressure [88], and, most pronouncedly in bigger and heavier animals like birds, their dorsoventral polarity also produces a lifting force – similarly to aircraft wings. Importantly, the adaptation of locomotor systems to life on land had most probably been preceded by the evolution of benthic locomotion, which also requires a 2D movement, very similar to that required on land, and which, most probably, also goes together with dorsoventral polarisation. This is a clear example of the influence of physical forces on overall body symmetry and shape. Thus, since the link between locomotion and bilaterality seems to be evident, it can be argued that bilateral symmetry is optimised for physical forces in locomotion in the macroscopic world, i.e. it is ultimately formed by physical laws, at least to a significant extent. Other potential ultimate factors which favour bilaterality remain to be discovered. It could also be asked whether the body-scale bilaterality present in non-moving (sea anemones) or slowly moving taxa (mussels) confers evolutionary advantages, is due to phylogenetic inertia, is an admixture of the two, or is the product of currently unknown factors; however, this type of analysis would require detailed, taxon-focused investigations, which would go beyond the limits of the present paper.

What about the ultimate causes of radial body plans? The function of the overall radiality of cnidarians and echinoderms is explained by their sessile, drifting or slowly moving lifestyle [e.g. 78, 88]. The ordering of body parts according to this symmetry offers the ability to react to environmental forces in every direction with the same efficiency [1, 88]. Interestingly, a recent study has reported that following the amputation of a variable number of arms, the ephyra larvae of the jellyfish *Aurelia aurita* regenerate their radial symmetry, rearranging the remaining body parts without restoring the missing arms [91]. The process, called symmetrisation, is completed regardless of the number of arms lost, and without any obvious global organiser in the body: it is driven by muscular contractions, pointing out both the importance of mechanical forces as proximate form-shaping effects and the need to restore radial body symmetry [91]. According to the manoeuvrability hypothesis [88], however, the radial body form cannot allow such a fast and precise locomotion as the bilateral, as is clearly observable in nature (e.g. cnidarian and echinoderm locomotion). The convergence to the cylindrical form of endoparasites and burrowing worms – other groups of animals with radial external symmetry – has been proposed as the logical consequence of the fact that they live in a very dense substrate where locomotion favours body plans whose cross section area is minimised [88]; consequently, the cylindrical symmetry is optimised for their specific lifestyle and is shaped by physical forces. The decoupling of the external radial symmetry and the internal bilateral structuring of burrowing and endoparasitic worms [88] underscores the flexible use of symmetrical anatomical patterns in response to functional and physical requirements [1, 2]. Thus, it can be stated that the indirect cause of this symmetry, too, is to conform to the physical environment; i.e., it is optimised for physical laws – whether they be manifest in the sessile, the drifting or the burrowing lifestyle of the animal ([88]; see also ref. [1]).

## Conclusions

The idea that symmetry is mainly shaped by physical forces, has deep roots in time; however, with the advent of modern molecular biology, the molecular approach has taken the leading role in science. For example, a century ago, D’Arcy Thompson proposed that physical forces were involved in the generation of a series of symmetrical structures, such as microscopic cells, the eggs of birds (passing through, and so shaped by, the uniformly dispersed forces by the peristaltic contractions of the tubular oviduct), and the radially symmetrical cnidarians [90]. Since the publication of his book, numerous experiments have led to the same conclusion, as listed in previous sections of this essay. Nowadays, the time might have come to re-evoke the old, common sense logic, and re-synthesise knowledge on animal symmetry, explained not only by molecular factors but also by mechanical forces.

In summary, I think that instead of treating animal symmetry in general terms as, for want of something better, a combination of developmental canalisation and historical contingency, a more mechanistic view should be adopted. Any idea in which symmetry is mainly a genetic and developmental “burden” about which we do not really know why it changes in certain instances and why it remains the same for hundreds of millions of years, and which fails to explain why bilaterality is associated with a free-moving lifestyle in certain cases and why it is not in others, remains, in my opinion, unsatisfactory. In this concept, the whole story of animal symmetry is fragmentary, and the pieces of the mosaic are not held together by any coherent explanatory concept. Interestingly, however, the examples of symmetrical patterns of biological structures that turn out to be logically reasonable are justified by physical-type explanations.

Disentangling the question of what types of constraints, and to what extent, act on shaping the evolution of animal form, is an attractive problem. However, it seems that exact solutions to this puzzle do not exist in principle, given that we have neither the methods to analyse them in detail, nor any process which could serve as a control situation. Thus, any answer has to be necessarily speculative. The main types of constraints acting in evolution are classified into two main groups [92]. First, the mechanical-architectural and the functional constraints stem from structural-functional limitations and physical laws, and they only allow the formation of a subset of the theoretical morphospace. Second, the developmental and the genetic constraints originate from the non-random production of variants [92]. The analysis of the different involvement of these diverse constraint types in shaping morphological properties can be fruitful on minor time- and taxonomical scales, such as across orders or families. However, trying to explain symmetry across the whole of documented animal evolution only by developmental and genetic constraints, seems to be insufficient and misleading. This is also because symmetry is a basic property of the organisation of matter, and genetic and developmental constraints can only come into existence *after* mechanical-architectural and functional constraints have delineated the basic geometric features of biological structures. Regarding functional constraints, it has been shown that not all conserved phenotypes are the fruit of convergent evolution constrained by functional necessity; they may simply be frozen combinations on a local optimum of the fitness landscape, limited by unpassable valleys in the genotype space [93]. This most probably does not hold for symmetry, which frames every phenotype in animal evolution.

I propose a flexible concept of symmetry in which simple physical laws, through function, determine which of the symmetries will be expressed from an animal genome that encodes both of them. In such a mechanistic view, one does not treat as exceptional and incongruent such phenomena as why it is that an endoparasitic animal can have internal tetraradiality and a cylindrical external shape despite being a free-moving animal [94], or why the bilateral spine distribution of a sea urchin can be explained by the improved defensive function it confers on the animal, and not by efficient locomotion [95].

The following opinion about symmetry in animal evolution appeared 15 years ago, in a seminal paper: “As for the shapes of life, macroscopic forms are most likely to be multicellular and there is a finite set of simple geometries — such as those that dominated the early history of life on Earth (linear and branched filaments, cylinders and spheres) — that are likely to satisfy the constraints imposed by diffusion and biomechanics and that are therefore likely to be universal. But the evolution of motile, modular mega-organisms may be a different story. […] although some symmetrical body organization is likely of macroforms, there is no basis to assert that bilateral, radial or spiral forms were or would be inevitable.” [4]. In contrast to this view, I propose a unifying frame of thinking, according to which, the symmetries present in the diverse organisational levels of the animal body are mainly shaped by physical effects and, in this way, by functionality; thus, their appearance in animal evolution is inevitable. On the basis of the reasoning already presented, helical symmetry, synonymous to the “spiral forms” mentioned in the previous citation, is only expected to be present in lineages which conduct a sessile or slowly moving lifestyle, to serve protective purposes and to act as mechanical stabilisers, as seen for example in sponge skeletons [33].

Since overall spherical symmetry is suboptimal for the body plan of a macroscopic animal that has to deal with gravity and the physical challenges imposed by locomotion (such as drag; [88]), it is only radial and bilateral symmetry which can be deployed when constructing its body. It seems to be obvious that a profound inertia caused by the genetic canalisation of development is characteristic of the evolution of body plans, but, regarding only symmetry as a basic and omnipresent feature of body plans, I emphasise its physically determined character: speaking in terms of geological time, it seems very improbable that the explanation of the symmetry of the body plan or that of minor anatomical structures (such as biological tubes) should invoke developmental and genetic constraints. Bearing in mind (i) that symmetry is a ubiquitous feature of biological structures in every level of individual and infra-individual organisation, and also considering (ii) the limited number of practically possible symmetry types, (iii) the physical environment of Earth, (iv) the enormous amount of time for any potential change in the symmetry of body and transport systems, and (v) the capability of the animal genome to build both radial and bilateral symmetries, the idea of the determination of symmetry by physical forces further bolsters the concept that both radial and bilateral symmetries are necessary products of animal evolution [2, 88]. Thus, in my considered opinion, if the tape of life [96] was rewound and started again, the many detailed architectural patterns of animal body plans would probably differ from the actual patterns, but the basic symmetries characterising body plans and the many anatomical structures would be identical to those that we find today. Hopefully, our picture of animal symmetry will be further clarified when we will eventually be able to identify the ultimate causes behind the very origin of either radial or bilateral symmetry, long-sought answers to fundamental problems in evolutionary biology.

## Eugene Koonin

In this manuscript, the author strives to ‘demystify’ animal body plan symmetry by proposing that symmetry is shaped primarily by physical factors rather than functional adaptation. On the conceptual plane, I support this view because in evolutionary biology, a non-adaptive null hypothesis is generally preferable to any adaptationist ‘just so story’. Under the premise that it is this null hypothesis that has to be falsified before any functional/adaptive causes are even considered, I suppose, the article does what it is supposed to do. That said, there is very little concrete, let alone quantitative, argument here as how, specifically, physical factors produce symmetry. Furthermore, the previous work from the author (Ref. [87]) that is cited here as the best available account of bilateral symmetry evolution speaks of animal symmetry in terms of adaption that optimizes locomotion in a given (e.g. particularly dense) media. Surely, the adaptation takes this particular form because of the physical properties of the environment but isn’t this a salient aspect of any adaptation? Regrettably, I do not have the impression that direct and direct causes, and biological and physical factors are disentangled here in a satisfactory manner.

I believe the paper would gain a lot from a more specific description of the way physical factors shape symmetry. The best thing would be to provide actual estimates (even ballpark ones) of the effects of the forces involved. I realize that this is a tall order but any approximation woudl be valuable.

*I am grateful to Dr. Koonin for undertaking the review. I also admit that the paper lacks specific descriptions as to the precise extent physical factors determine symmetrical patterns in the animal body. However, please let me first underline that this hypothesis paper tries to give a general framework for thinking about symmetry, and not to offer exact explanations for individual cases for the specific animal taxa. Furthermore, to be able to give even approximate numbers for these intervals, the concrete values of the forces involved should be individually measured (and published as research articles), which, I think, exceeds the scope of the present paper. However, I am open to conducting further investigations; in this case, please, give more specific details on how to proceed.*

## Zoltán Varga

### General comments

The Author tries to provide a unified “mechanistic” solution for the origin of symmetry of the animal body. His explanation offers a “cutting of the Gordian Knote” with the key words: “Animal genome should be regarded as a system which can construct both the main symmetries – radial and bilateral – *simultaneously*”. The paper is rich in original ideas, therefore it is worth for discussion and thus, also for publication, although I cannot agree with some of its basic ideas. As a consequence, I suggest a careful revision of the paper but I am also waiting for the objections of the Author in his answers on my criticism.

I think there are two basic flaws of the paper. The first is more philosophic, the second more phylogenetic (incl. EVO-DEVO).

(i) The survey of causality is incomplete! The general “bauplan” is constrained by the life style, e.g. benthic errant, benthic sessil, pelagial planctonic, etc. In details, e.g. blood vessels, digestive channel etc. these constraints are directly connected with the function. However, while the “bauplan” can be constrained by phylogenetic ancestry – i.e. more by some “causa finalis”, the second is the consequence of more direct, proximal “physical” factors: “causa efficiens”. These are insufficiently disentangled in the paper.

*First of all I would like to thank Dr. Varga for having undertaken the work of reviewing the manuscript.*

*Please let me note first, that according to the logic presented in the essay, both the whole body and the infra-individual level structures act as biological entities reacting to the forces of their environment. Furthermore, both are built on the basis of genetic programs, which follow a linear order of activation. Naturally, the core of the genetic programs –* i.e. *the initially activated “kernels” of the GRNs which mark out the basic bauplan – are the most stable ones in evolutionary terms,* i.e. *the most constrained phylogenetically. However, in no way does this imply that the whole body should not conform to physical factors, and that the mechanistic view could not also be adopted for the general bauplan. This means that even though in the case of minor anatomical structures the physical forces may much more easily be identified as the causa efficiens, both the body-level and the infraindividual symmetries can well be constrained by causa finalis, (even if, for example, for the bilaterally symmetrical body this is not so obvious at first sight), which means the aim of both is to fit the physical environment.*

(ii) If the general “mechanistic” paradigm of the Author would be valid, he should be also able to refuse the existence of a general bilateral “grundplan” of all triploblastic animals (see: “Urbilateria theory” which is underpinned with the whole evolution of the HOX-PARAHOX genetic system). I think, this basic problem remained unsolved and also undiscussed in the paper.

*As my answers below will try to highlight, the rejection of this theory is not necessary. What I propose only requires a shift away from the view that sees the whole of morphological evolution as the manifestation of genetic programs passing from generation to generation. In this aspect, it is mainly, or only, the genetic information which constrains the individual bodies so that they develop in a specific order, and it is only mutations and other – also stochastically acting – genetic effects which produce the variability on which natural selection operates. Simply put, evolution of form springs from genetic processes. This is also true but is only one side of the picture. I think that even if genetic processes do have their own laws, the organisms in which the genetic programs are manifested have to fit physical effects, otherwise non-conforming forms will be ruled out from evolution. Thus, morphological evolution has to follow genetic processes, but genetic processes have to follow physical effects.*

*Development, in itself, is mainly a strict manifestation of a genetic algorithm, and even the direct action of physical forces is largely hidden: intricate and meticulous experiments are necessary to see how physical effects work during development – but now this has also been widely acknowledged, as it is evident from the many works listed in the paper. In my opinion, evolution is, however, not simply the “sequence of unfolding of genetic programs”: the effect of physical forces should also be added to the genetic story – even if many times they can only be educed. Furthermore, I suggest that, in terms of the evolution of symmetry, they are the guiding factors.*

*If I am right in perceiving the reasons behind the objections, their main source was that several of the statements I made were inaccurately formulated, and sometimes not clearly defined, either (*e.g. *“hierarchy”). I have tried to make them more precise, and so I hope now the message is more effectively conveyed to the readers.*

## Detailed comments

### Abstract

I cannot agree with the thwo basic sentences below:

Row 15: “Animal genome should be regarded as a system which can construct both the main symmetries – radial and bilateral – *simultaneously*; and that the expression of any of these depends on functional constraints.”

– Oppositely, I think the basic “story” of animal phylogeny is the loss of the radial bauplan as a consequence of the triploblastic organisation. Triploblast organisation is a “stage of no return” both in the phylogeny and ontogeny of Animalia.

*Thank you for pointing this out, the sentence was not accurate. It has been modified to: “animal genome, on large time scales, should be regarded as a system which can construct both the main symmetries – radial and bilateral – simultaneously; and that the expression of any of these depends on functional constraints”.*

Row 18: “Current theories explain biological symmetry as a pattern mostly determined by phylogenetic constraints, and more by chance than by necessity.”

- The second part of the sentence is not the consequence of the first. Otherwise I fully disagree with the second statement since I think that the phylogenetic constraints are “necessities” (I carefully studied not only Carroll 2001 but also 2008!).

*You are right: the sentence summarises two ideas coming from two different sources. The first part is expressed by García-Bellido 1996, the second by Carroll 2001. Unfortunately, in the abstract references cannot be used, but the same information, now with citations, appears right in the first paragraph of the “Introduction”, and hopefully clarifies the sentence, both parts of which I will try to refute later in the paper.*

## Introduction

Row 33: “The concept of the body plan can be defined as an ontogenetic pattern-organising algorithm, thanks to which the body develops in a specific order.”

I think, the problem of symmetry in the general “bauplan” vs. functional details should be clearly disentangled.

*The distinction between whole body symmetry and regional level symmetry is dealt with later in the Introduction section; please also see my answers which follow below.*

Row 43: “The evolution of animal form is mainly caused by the changes in the regulatory genes of the genome.”

– The Author tried to refuse this statement. However, it was essentially NOT refused in the paper, therefore one should ask whether the two approaches could not be complementary: the genetic/phylogenetic for the bauplan, the “mechanistic” for details (functional constraints).

*It might seem that I tried to reject the statement cited above, but I did not. Conversely, this notion supports my view. If the changes in animal form are due to changes in the GRNs, then it is important to study the fundamental and general properties of the operation of GRNs. And since these are mosaic both in terms of their evolutionary history and their functioning, it may be inferred that there is no essential and compulsory hierarchy between the diverse GRN modules from which the body is built up, in terms of symmetry. For example, it is not mandatory that every part of the body should be bilaterally symmetrical only because the basic organisation of the whole body follows that order, governed by the first activated GRN subcircuits. Later activated circuits may express another, different symmetry type if that serves the animal.*

Row 50: “In this view, in terms of genetic programs, the difference between the establishment of the basic geometrical features of the body plan, the specification of progenitor fields for developing organs, and the formation of tissue-level details, is only a difference in the timing of subsequently activated GRN modules.”

- This statement must be questioned since these (body plan, organogenesis, tissue-level details) are hierarchically organised (nested hierarchy!), therefore the difference is surely not only the timing!

*Please see my answer to the following objection.*

Row 50ff: “In accordance with these general and basic properties of GRNs, it has recently been proposed that the determination of the symmetries in diverse levels of the body plan should also be regarded as a question of a different timing, not as the manifestation of a real hierarchical relationship [2, 13]”.

- See my objection above!

*In the sense of biological organisation, the formation of the diverse body parts is hierarchical. The genetic program, itself, is also hierarchically organised in the sense that the order of kernels and the outer shells of the GRNs cannot be changed or mixed. However, the GRN subcircuits are separate from each other, and their activation follows a linear path. In this linear code, the subunits are not, of course, independent from each other, but have quite a clear autonomy: what is happening in the later operating subcircuits is not directly influenced by the previous subcircuits. Thus, considering only the symmetry of the diverse structures, there is no evidence to claim that all symmetrical patterns must follow the firstly established,* i.e. *general symmetry of the body.*

*I think the basic reason my reasoning was incomplete and gave rise to potential confusion in the reader, was the lack of a clear definition of the word “hierarchy”, since this word has also been used in different senses, even by me. Now the sentence has been completed and reads:*

*“In accordance with these general and basic properties of GRNs, it has recently been proposed that the determination of the symmetries in diverse levels of the body plan should also be regarded as a question of a different timing, not as the manifestation of a real hierarchical relationship* [2, 13] *(hierarchy is defined here as the capability of a sub-program to directly control or overwrite another sub-program).” (Rows 62–63.)*

*I hope with the specification of the word “hierarchy” the problem has been solved and the text has been made clearer.*

Row 50ff: In this view, it can be said that the overall symmetry of the body plan is not *the* symmetry of the animal, since the symmetries of minor body parts also have to be taken into account when speaking about body plan symmetry.

**- I believe that in terms of symmetry surely NOT!** In this statement the nested hierarchy of the body organisation is completely forgotten.

*Thank you, the sentence has been changed by inserting the word “only”, as follows:*

*“In this view, it can be said that the overall symmetry of the body plan is not the only symmetry of the animal, since the symmetries of minor body parts also have to be taken into account when speaking about body plan symmetry.” (Rows 63–65.)*

Row 62: “The overall bilateral body symmetry of bilaterians is combined with regional radial symmetry (such as that of the eye balls, and the biological tubes of the circulatory, respiratory, urogenital and glandular conducting systems). Thus, it has been suggested that the animal body can be regarded as a flexible system in terms of symmetry, capable of constructing either bilateral or radial symmetry [2, 13]”.

- Both statements are true but the second one cannot be concluded from the first, since the bilateral body symmetry is a higher level of organisation and more than the sum of the “flexible” elements!

*I think with the previously described modifications this sentence also acquires sense; however, it has been further refined, as follows: “Thus, based on theoretical considerations regarding the functioning of the GRNs described above, it has been suggested that the animal body can be regarded as a flexible system in terms of symmetry, capable of constructing either bilateral or radial symmetry* [2, 13]*, be they manifested either in the general body plan or in infraindividual structures.” (Rows 71–74.)*

Row 76: “Mathematical modelling has suggested that merely by coupling two signalling pathways acting in epithelial morphogenesis, under certain parameters the process “automatically” leads to the formation of very basic body plans with either radial or bilateral symmetry [14] (see also [1]). This indicates that the basic molecular organisation required for building any of the two symmetries is relatively simple.”

- I think this argumentation is wrong! The basic problem is the modular organisation, i.e. the segmentation which will be expressed or not! The modular organisation IMPLIES “an sich” the bilateral symmetry or even the asymmetry. It means that the triploblastic organisation is an essentially new “environment” both for the ontogeny and phylogeny of the “bauplan”.

*I agree that the triploblastic organisation offers a brand new “field of possibilities” for animal body plans to evolve. However, I think this, in itself, does not contradict the results of the modelling reported by Frederick W. Cummings (2006, Int. J. Dev. Biol.), since a simple, basic bilateral symmetry can also arise without segmentation, thus the genetic machinery required for segmentation can be embedded in another genetic program which already builds bilateral symmetry.*

*Morphogenesis and physical forces*

Rows 112 to 137: “Similarly, Coulombre and co-authors suggested that the pigmented epithelium of chicken embryonic eyes increased in area in response to tensile forces acting in its plane [31]. Later on, Desmond and Jacobson pointed out that the correct enlargement and shaping of the chick embryonic brain was dependent on the mechanical force produced by cerebrospinal fluid pressure…”

- Several examples are mentioned here which demonstrate the direct influence of physical constraints. Surely, the Author is right that physical environment must shape the morphogenetic processes. All mentioned examples, however, refer on details of organogenesis and not on “groundplan” level processes like bilateral symmetry vs. asymmetrisation of the body. E.g. it would be difficult to imagine the process of the helicoid asymmetrisation simply in terms of physical forces.

*You are right to observe that this part of the text only deals with the regional level effects of physical forces, and its aim is to highlight the fact that genes and morphogenes cannot be sufficient to explain morphogenetic events. However, as emerges from the following passage “Mechanical forces and the overall body symmetry: the establishment of symmetry in the animal body and the indirect causes of body plan symmetry”, physical forces seem not to directly influence the formation of groundplan level symmetries, but they do seem to act as selective agents, to which the body symmetry has to conform. Asymmetrisation can thus always be present when symmetry is not constrained by locomotion, or by physical forces in general, so it does not necessarily have to be under a direct influence of physical forces; what allows asymmetrisation to develop is rather the absence or reduced importance of the effect of physical forces regarding the given structure.*

*The title of this section has been changed to “Influence of mechanical forces on morphogenetic processes”, so as to be more expressive.*

Rows 230ff: *“*Mechanical forces and the overall body symmetry: the establishment of symmetry in the animal body and the indirect causes of body plan symmetry*”– This chapter is the most problematic part of the paper.*

Row 233: “Overall body symmetry arises at the beginning of development, from the original spherical symmetry which forms by the physical effects of the microscopic world (the eventual internal asymmetry of the egg, given for example by yolk distribution is, naturally, permitted, since its internal environment is not in direct physical interaction with the outer world). In this realm, before tissue stabilisation, aggregates of motile and mutually adhesive cells essentially behave as liquids, and their shape changes are governed by surface tension via the diminution in the adhesive-free energy of the cell population”.

- Differences in yolk distribution occur independently in phyletic lines both with radial and spiral cleavage (see: discoidal cleavage, e.g.). The phylogenetically most important event is, however, the basic divergence between radial and spiral cleavage – latter occurring in triploblastic animals only! This is usually connected with an early determination of blastoderms and tissues, and this is the very first “break of symmetry” in Lophotrochozoa – I think from this point there is “no return to radial symmetry” in Bauplan!

*Please see my answer below.*

Row 241: “In this environment, while the dividing zygote becomes a morula and then a blastula, the spherical symmetry that is established is a simple reaction to the physical environment: cells spontaneously take a spherical form, minimising their total surface area, and this shape is also the simplest geometrical arrangement which responds to equally distributed forces.”

If this statement would be valid, how could we explain the emergence of the spiral cleavage!?

*Thank you for pointing this out, my phrasing was confusing here. I would like to highlight the emergence of the blastula as a spherically symmetrical structure, to emphasise that the symmetry of the blastula stage is the symmetry from which the body symmetry forms, and that there is no sense in speaking about preceding phenomena such as yolk distribution and cleavage. By referring to the uneven yolk distribution I wanted to point to the importance of the interaction between the environment and the external layer of a biological structure, but I admit that the formulation of the whole idea was obscure and misleading. The part in parentheses has been omitted and the later sentence has been simplified and refined: “With the formation of the blastula, the spherical symmetry that is established is a simple reaction to the physical environment…” Please see rows 244–248.*

Row 253: “Thus, nature adopts an elegant way to establish radial or bilateral body symmetry: in the first step, the most perfect – spherical – symmetry is generated, and then it is “flawed” to create radial or bilateral symmetry.”

- This very nice formulation should be underpinned by some basic processes of “bauplan” morphogenesis, however! The next constraint of bilateralisation is the formation of mesoderm and coelom (both in phylogeny and ontogeny)! These facts remain unexplained in the paper! The physical constraints of “radialisation” are demonstrated in some cases but these are “individual” episodes without phylogenetic significance.

*The following sentence has been added to make the argument more precise: “This process is accompanied, and also effectuated, by morphogenetic events such as the formation of germ layers: in radially symmetrical taxa, the ectoderm and endoderm are generated, to which the mesoderm and the coelom are added in bilateral animals.” (Lines 256–258).*

Row 337: “However, searching for developmental and genetic constraints while examining symmetry across the whole of documented animal evolution seems to be a vain endeavour. This is also because symmetry is a basic property of the organisation of matter, and genetic and developmental constraints can only come into existence *after* mechanical-architectural and functional constraints have delineated the basic geometric features of biological structures.”

- “Examining symmetry across the whole of documented animal evolution seems to be a vain endeavour”- I do not agree! The bilaterisation is a general trend, often connected with secondary asymmetrisation – e.g. in insect external genitalia controlled by sexual selection.

*I am sorry for the wording, which may have led to misunderstandings. The sentence has been refined: “However, trying to explain symmetry across the whole of documented animal evolution only by developmental and genetic constraints, seems to be insufficient and misleading.” (Rows 379–381).*

Row 356: Second, the appearance of a single cell stage – the egg – in the life cycle of multicellular organisms has been proposed as a necessary step in evolution since it increases the evolvability of the organism, and also reduces the probability of intraorganismal cell-cell conflict [95]. Thus, the egg itself is not inevitably necessary for the multicellular organism because many cells should and could develop only from a single cell, but is rather a versatile adaptive tool for evolvability and for the exploration of a diversity of life strategies.

- Misundertanding of the basic animal life cycle!

*I am afraid I do not understand why this would be a misunderstanding. As argued by various authors (*e.g. *Wolpert L, Szathmáry E. Nature 2002; 420:745; Newman SA. J Exp Zool (Mol Dev Evol) 2011; 316:467–483), it is theoretically possible to also “start” a lifecycle from multicellular scenarios, but the single cell stage is evolutionarily advantaged over multicellular stages. However, while I was writing the answer to the concern raised by Dr. Manuel (please see below), whose objection referred to another part of this subsection, I had to admit that the whole argumentation on early embryonic events does not essentially affect the main line of thinking of the article (either in a supportive or a contradictive sense), and so it should be left out of the text. The remaining part has been inserted into the last section of the paper.*

Row 380: “We do not really know why it changes in certain instances and why it remains the same for hundreds of millions of years, and which fails to explain why bilaterality is associated with a free-moving lifestyle in certain cases and why it is not in others, remains, in my opinion, unsatisfactory.”

- Unfortunately I cannot agree with this conclusion since: (i) the various forms of bilateral animal symmetry have been emerged on the basis of triploblastic organisation, therefore (ii) there is given a basic line which “remains [essentially] the same for hundred millions of years. This common basis of bilaterality is independent from the actual style of life, the latter only can modify either the “whole” (see: pseudo-radial external symmetry in Echinodermata) or some details (“tubular” organs) which do not influence the “bauplan”.

*I am sorry, but I see some conflict in this reasoning and I have to disagree to some extent with this opinion. That bilateral basic organisation is a long-lasting pattern in body plan evolution is a fact, but it is not in contradiction to what I expressed in the statement in question, because it is only descriptive information, not explanatory. The external radiality of Echinoderms may be called pseudo-radial external symmetry, but in fact it is just a difference of terminology, since the latter expresses the idea that the external radiality is superimposed on a basic bilaterality. But, again, this is only descriptive information, not explanatory. The tubular organs do not influence the whole-body symmetry, but the manuscript did not state this either: minor organs have their own symmetry, since the animal genome is capable of producing it even if the basic body plan is bilateral. Conversely, some bilaterally symmetrical structures are expressed in the cnidarian body even if the whole symmetry is radially symmetrical. So far, this is only a description of the body patterning of diverse animal lineages. However, the view that these symmetries do have their function in nature –* i.e. *their basic geometrical features have to conform to physical forces – offers an explanation for their evolution. In this aspect, one can clearly see that even if the basic body organisation is bilateral, the form of burrowing animals, endoparasites and drifting animals converges towards radial symmetry. They may not be “perfect” in terms of human abstract geometry, they may be superimposed on a different basic body scheme, they may be only external (the external form of Echinoderms) or only internal (tubular organs in bilateral bodies), but their intimate connection to physical forces cannot be overlooked, and so some explanative power can emerge here. I do not propose to negate or subvert previous knowledge on animal evolution, I only propose to complete it.*

## Michaël Manuel

This paper addresses symmetry in the animal body by adopting a very broad perspective and underscoring the role of mechanical/physical forces both as a direct cause of the establishment of symmetry during development and morphogenesis, and as its main “indirect cause” (= the cause which gives a selective advantage). The main consequences of these considerations are that body symmetry arises by necessity given physical laws and that a general understanding of the significance of the main symmetry types of organisms is possible. This kind of exercise is necessarily rather speculative, but the author builds upon a rich and documented corpus of empirical evidence (particularly in support of mechanical forces as a proximal driver of symmetry establishment during morphogenesis), and all things considered I see this paper as a useful, sound and convincing contribution. Understanding the significance and underlying causes of organismal symmetry is an important issue that has often been neglected or only superficially dealt with in the past. The text is very well written and is generally easy to follow. However, I have a few concerns that should be considered while revising the manuscript.

### Major recommendations

First, the abstract does not help much to understand the general message of that paper. This is in part due to the use of the term “indirect cause” (line 21) without any explanation. This term is not self-explanatory. I think the abstract should express and summarise in a much clearer and more expanded way the main idea(s) pushed forward in the paper.

*First of all let me express my gratitude for your work.*

*Thank you for the observation. The abstract has been expanded, and the words “direct” and “indirect” have also been clarified by the terms of “proximate” and “ultimate”, which explain their significance better in an evolutionary context.*

In the text, the definition of “indirect cause” should appear earlier and be better emphasised.

*To the first paragraph of the Introduction, the following definition has been added: “In this paper, the factors that directly shape biological patterns will be referred to as direct or proximate causes, while the factors which give a selective advantage to the given form –* i.e. *they explain what that form is good for – will be termed as indirect or ultimate causes.” In addition, at some points, the term “indirect” has been changed to, or complemented by, “ultimate” (rows 232, 324).*

The paper is largely written as if the main idea was entirely novel, but in fact the proposition that physical forces are the main driver of body symmetry is not new (although in the past it has remained quite marginal). Notably, I have been surprised not to see D’Arcy Thompson’s book “On growth and form” (1917, Cambridge Univ Press) among the references. The author should review this book and analyse to what extent his own ideas overlap with those of D’Arcy Thompson or depart from them.

*His wide-ranging thoughts are referred to in the text, regarding gravity, physical constraints, and the radial symmetry of diverse structures. See rows 297 and 349–358.*

There is a major flaw affecting one of the most pivotal parts of the paper and the corresponding figure. This problem can be easily corrected, without weakening the argument (on the contrary, full consideration of this issue will strengthen the demonstration). Panels B and C in Fig. 3 are said to represent a bilateral body and are intended to illustrate how bilaterality is important to optimise directional locomotion. However, none of these two drawings represent a bilateral morphology. I invite the author to look at his Fig. 3b and c to realise that in both cases there are two symmetry planes: a vertical one but also a horizontal one. Thus, these two drawings represent biradial morphologies, not bilateral ones. This is not a question of playing with the words, as biradiality and bilaterality are fundamentally different (single polarity axis in the former vs. two polarity axes in the latter). To say it in a different way, the problem is that Fig. 3b and c do not integrate any dorso-ventral polarity (even the “appendages” in Fig. 3c are represented without any dorso-ventral polarity!). Figure 3b could be let as it is (but clearly stating in the legend and the text that this represents a hypothetical biradial condition associated with directional locomotion), but at least Fig. 3c should be modified as to render it truly bilateral.

*Thank you very much for the observation, both the figures and the legends have been modified.*

This problem significantly impacts the reasoning presented in pages 12–13, which consists in an explanation of the functional significance of bilaterality, in the context of directional locomotion. Here there is a detrimental lack of consideration of preferential orientation with respect to gravity, which in combination with directional displacement and morphological differentiation between the forwards and rearwards poles, fully accounts for bilaterality in shape. Directed locomotion and antero-posterior polarity without definite orientation with respect to gravity exists in nature and is not associated with bilaterality. For instance, cnidarian planulae do swim directionally, they do have definite anterior and posterior poles, but they have no dorsal/ventral sides. When they swim they constantly rotate around the oral/aboral axis (like a spinning top), and correlatively, they are not bilateral (but cylindrical). This example shows that contrary to what the paper says, directional locomotion per se does not require bilaterality; you need to consider in addition definite orientation with respect to gravity (and/or to the substrate). This important parameter should also be incorporated into considerations about the mechanics of locomotion in first half of page 13. Actually, this is done for benthic locomotion (2D movement), and very incidentally for 3D locomotion in the air (line 293). What is lacking is a consideration of the importance/usefulness of bilaterality (that is to say, not only antero-posterior polarity, and the lack of multiple radial structures, but also dorso-ventral polarity) in the context of directional swimming (3D locomotion in water). Here, I think the author is wrong when considering that hydrostatic pressure (Archimede’s principle) is sufficient in water to counteract gravity (line 294). Aquatic organisms are denser than water (except some planctonic organisms that have special devices such as cavities filled in with gas or lipids, to render them less dense than water), so for macroscopic organisms, efficient swimming requires the production of a vertical force (in addition to the pushing force or thrust) to counteract weight. This force is called lift. As a suggestion, I believe that this part of the paper would benefit from an analogy with the aerodynamics of airplanes. Indeed, airplanes are bilateral in design and this bilaterality is inherently associated with how lift is generated when the airplane moves along its fly path in the air, at a sufficient speed (for a good introduction to the physical forces acting on an airplane and how lift is generated see chapter 4 in the US FAA “Pilot’s handbook of aeronautical knowledge”, downloadable on the FAA website). Particularly relevant to this discussion is the fact that lift production by the wings involves some difference in profiling between its upper and lower surfaces (= dorso-ventral polarity). The airplane moves in the air but the same rules apply to any kind of body moving in a fluid. I think that accounting for the necessity of a lifting force while swimming will fully explain, in addition to the argument of reduced sideway resistance (also true for the airplane: multiple radial wings would increase drag dramatically), why bilaterality is required (or at least, helps much) in this context—whereas the present demonstration is not fully convincing (for the obvious reason that the idealised forms underlying the discussion, i.e. those of Fig. 3b and c are NOT bilateral). Of course, there are other potential advantages for swimmers in keeping constantly the same position with respect to up and down (e.g., in terms of perception of their environment).

*Thank you for pointing out the question of polarity with respect to gravity, which has been unworthily neglected. I think the lifting force stemming from dorsoventral polarity should only come into play when the body size oversteps a threshold (without assessing exact parameters), because with greater dimensions the viscous forces gradually become less and less important in locomotion. Nevertheless, it is a very important component of the discussion of bilaterality and locomotion. This criticism was very helpful in allowing me to develop a deeper understanding of the problem. The following part has been added to the text: “To complete the picture, it is important to mention the role of gravity in the determination of dorsoventral polarity* [1, 90]*. To produce sideways turning forces it is enough to have a laterally flattened body (Fig.* *3* *b),* i.e. *biradial symmetry. However, in dimensions characterised by even greater Reynolds numbers, the viscosity of the fluid will be not enough to hold the body, and hydrostatic pressure will not be able to fully counteract gravity. In this realm, the dorsoventral polarisation, which produces a different profiling of the dorsal and ventral sides of the body, and, most importantly, of the appendages, will help to produce a lifting force. This dorsoventral polarisation leads to the advent of the second polarity axis, thus reducing the number of the two symmetry planes of a biradial body to one, generating a bilaterally symmetrical body.” (Lines 296–305.) Other sentences have also been enriched to incorporate this information; please see rows 308–310 and 313.*

To end with this part of the paper, I have two additional less crucial (but not completely unimportant) concerns:

- this discussion is very much “Bilateria”-centric, as it focuses exclusively on directional locomotion. However, there are among animals other forms of body-scale bilaterality that have nothing to do with locomotion, for instance the bilateral symmetry of many anthozoan polyps (see discussion in ref. [1]). Beklemishev (ref. [84]) also gives the example of a hydrozoan whose polyps are placed at the margin of the tube of a polychaete worm; they have two tentacles inserted towards the tube opening and thus are bilateral (whereas completely immobile). Furthemore, even within bilaterians we can observe that very overt forms of bilaterality can persist in non-mobile taxa (think for example about the body design of a mussel and how it relates to its sessile biology). This means that bilaterality in addition to its superiority for directional swimmers also has advantages in other lifestyles, and in some cases (e.g. mussel) these are clear instances of exaptation.

*Both the body-scale bilaterality of cnidarians and that of slowly moving taxa are interesting puzzles on which, however, I am somewhat reluctant to take a stand, because I think, too much speculation is needed if one wants to give a brief yet reasonable opinion. These designs might, for example, be simple variations to explore a niche range. In this conception, the body plan symmetry can depart from the typical designs of the mother taxon if that is not directly disadvantageous. I think that in those groups where precise and fast locomotion is not present, organisms have the opportunity to explore a range of possible geometries – see, for example, the symmetry of the biradial Ctenophores: they are not radially symmetrical as other tentacled sessile or drifting hunters are, but they are close to it. Similarly, a slight bilateral organisation of anthozoan polyps allows the animal to perform essentially the same functions which would have also been allowed by a strictly radial organisation: they are not radially symmetrical but the tentacle disposition is close to it. In molluscs protected by shells, the symmetry may depart from the bilateral; see, for example, snail shells which, following a simple algorithm to produce a coiled arrangement, can both accompany the growth of the animal and give a continuous defence to it; all possible because they are freed from the bindings imposed by quick locomotion. In mussels, the bilateral symmetry can well serve an effective, closable protective shell rather than being related to an efficient locomotion. However, all these variations of the major symmetrical designs would deserve more detailed surveys focusing on the given taxa, based on comparative anatomy and genetic analyses; I think the present paper cannot assume these lines of investigation.*

*The following part has been added to the text: “It could also be asked whether the body-scale bilaterality present in non-moving (sea anemones) or slowly moving taxa (mussels) confers evolutionary advantages, is due to phylogenetic inertia, is an admixture of the two, or is the product of currently unknown factors; however, this type of analysis would require detailed, taxon-focused investigations, which would go beyond the limits of the present paper.”; see rows 318–323.*

*A previous sentence has also been completed by inserting “(on the presumptive evolutionary advantage of the internal, bilaterally symmetrical structures of cnidarians, see ref.* [78]*).”, in rows 230–231.*

- the author relies on abundant self-citations when accounting for the functional properties of the symmetry types (ref. [87]), but this has also been discussed in detail by other authors (notably ref. [1]), which should be acknowledged.

*Thank you, this has been corrected in lines 327, 344 and 346.*

Finally, I found the whole “Canalisation and constraints” section (p. 14–16) weaker than the rest of the paper. Notably, the statement “searching for developmental and genetic constraints while examining symmetry across the whole of documented animal evolution seems to be a vain endeavour” should be more strongly justified to be convincing. This type of constraint is said to be relevant at lower-level taxonomic scales (up to families and orders), but I do not see why they would not also exist at least up to the phylum level (for example, in echinoderms, cnidarians…). The second half of this section (about variability/conservation in early developmental stages) is very weak, not only because of the lack of concrete examples, but more critically because it starts by presenting as a widely admitted fact that early development should be highly conserved. However, it has been recognised for a very long time (even in the 2nd half of the 19th Century) that the earliest stages of embryonic development are strongly variable, and more recently it is exactly this idea that is conveyed by the model of the “phylogenetic hourglass”, resurrected and popularised notably by D. Duboule in the mid 1990’s.

*The cited sentence has been modified and hopefully made more precise: “However, trying to explain symmetry across the whole of documented animal evolution only by developmental and genetic constraints, seems to be insufficient and misleading.” (lines 379–381)*

*I apologise for the second issue: the sentence was inaccurately worded, mixing two different things (namely, the intuitive view regarding the first foundations of a structure in general, and the widely known hourglass model). However, the more deeply I considered my answer to this criticism as regards the comparison between the different models for embryonic conservation (and mathematical approaches), the more clearly I had to realise that the argumentation on early embryonic processes will not actually provide sufficient support for the main line of the reasoning of the paper, because the question of the diversity of early embryonic developmental strategies to adapt to a wide range of niches does not, in principle, either bolster the flexible use of symmetries in the animal body, nor contradict it – thus the argument will still remain necessarily weak. Therefore, I decided to remove this part of the section, and merge the remaining part with the Conclusions section, where it fits well. Thank you for pointing out this problem.*

### Minor recommendations

- p. 18, lines 405–406: “it is only radial and bilateral symmetry which can be deployed when constructing a macroscopic body”. This is not true; there is at least a third fundamental symmetry type that this paper overlooks, namely helicoidal symmetry. This is the fundamental symmetry type of the body plan of terrestrial plants (and many plant structures, such as flower, pine cones etc.), which have macroscopic bodies. In metazoans, helicoidal symmetry is uncommon but not inexistent (whole skeleton symmetry of some hexactinellid sponges; see also the recent interpretation of the ctenophore body plan as presenting elements of helicoidal symmetry: Dunn et al. 2015 TREE, 30:282–291).

*I am sorry, maybe the sentence could give grounds for a misunderstanding: the sentence speaks about, and so is only valid for, the body of macroscopic, moving animals. However, the sentence has been modified, as follows. “Since overall spherical symmetry is suboptimal for the body plan of a macroscopic animal that has to deal with gravity and the physical challenges imposed by locomotion (such as drag;* [88]*), it is only radial and bilateral symmetry which can be deployed when constructing its body.”*

*The following sentence has also been inserted in the Conclusions section (rows 407–411): “On the basis of the reasoning already presented, helical symmetry, synonymous to the “spiral forms” mentioned in the previous citation, is only expected to be present in lineages which conduct a sessile or slowly moving lifestyle, to serve protective purposes and to act as mechanical stabilisers, as seen for example in sponge skeletons* [33]*.”*

- Figure 2: on the right, the BMP gradient is represented with the maximum at the ventral side. This is the situation in chordates, but in all other bilaterians the maximum is towards the dorsal side. It would thus be preferable to have the BMP gradient the other way around in this figure. The legend could include a note to say that the BMP gradient is oriented differently in chordates vs. other bilaterians.

*Both the figure and the legend have been modified.*

Minor issues:

- p. 4, line 62: I don’t understand why the pharynx is cited as an instance of regional-level bilateral symmetry in medusae (the other examples are OK).

*If the pharynx contains two syphonoglyphs, the symmetry becomes biradial, but when it contains one syphonoglyph, there is only one symmetry plane, and the symmetry is bilateral. It is true that the pharynx is, therefore, in not always bilaterally symmetrical, but I did not develop this topic in detail because the sentence only serves an illustrative goal. If you consider it is inappropriate, this example could be left out.*

- p. 6, line 119: “square-formed” do you mean “square-shaped”?

*Yes, thank you, it has been modified to square-shaped (now line 125).*

- p. 18, line 424, I do not understand the meaning of “ultimate causes” in this sentence.

*A definition has been added to the end of the first paragraph of the Introduction, and “ultimate” only refers to the origin of symmetries, since this question is still not fully explained.*

## Abbreviations

AP: Anteroposterior
BMP: Bone morphogenetic protein
DV: Dorsoventral
GRN: Gene regulatory network
Wnt: Portmanteau of *Wg* and *int* it means “Wingless-related integration site”
